# Unraveling the genetic potential of Indian rice germplasm for reproductive stage drought tolerance

**DOI:** 10.3389/fpls.2025.1454299

**Published:** 2025-07-18

**Authors:** Santhiya Subramanian, Pushpam Ramamoorthy, Subramanian Alagesan, John Joel Amalraj, Raveendran Muthurajan, Senthil Alagarsamy, Muthuramu Sengalan, Suresh Ramalingam, Pravin Kumar Kathiresan, Parthiban Thathapalli Prakash

**Affiliations:** ^1^ Centre for Plant Breeding and Genetics, Tamil Nadu Agricultural University (TNAU), Coimbatore, India; ^2^ Centre for Plant Molecular Biology and Biotechnology, Tamil Nadu Agricultural University, Coimbatore, India; ^3^ Department of Crop Physiology, Tamil Nadu Agricultural University, Coimbatore, India; ^4^ Agricultural Research Station, Tamil Nadu Agricultural University, Ramanathapuram, India; ^5^ Rice Breeding Innovations Department, International Rice Research Institute, Manila, Philippines

**Keywords:** rice, drought stress, mixed models, multi-environment BLUP, multi-trait selection index

## Abstract

Climate change poses a serious threat to future food security on a global scale. Drought is the most challenging abiotic stress, limiting rice production in rainfed rice ecosystems. Therefore, an experiment was designed under three distinct environments, non-stress irrigated condition, managed stress condition (Rainout shelter) and natural stress condition (Target Population of Environment) involving 500 Indian rice germplasms. The study aimed to assess the genetic differences, drought tolerance behavior and to identify potential drought-tolerant donors for climate-resilient drought breeding. The results revealed that yield-attributing and physiological traits were affected by drought stress, resulting in a grain yield reduction of 64.30% in managed stress condition and 68.12% in natural stress condition. A high heritability estimate for most of the traits under non-stress and stress conditions indicates that selection for grain yield under stress conditions can be done with the same precision as in non-stress condition. Mixed linear model revealed a considerable genetic variation for yield and yield-attributing traits. The correlation between the environments was found positive for the traits studied. Multi-environment trait association revealed that panicle weight, spikelet fertility and number of productive tillers per plant are the key pre-breeding traits for grain yield improvement under drought. Multi-environment analysis identified 47 accessions with yield superiority over the drought-tolerant checks. Multi-trait selection index reaffirmed that RL 4167, RL 6361 and RL 4131 are drought tolerant and high yielding under multi-environment analysis. Therefore, the promising high yielding accessions *viz*., RL 4167, RL 6361, RL 4131, RL 27 and RL 6298, thus identified, could serve as potential donors for grain yield improvement under drought stress.

## 1 Introduction

Global food security is being affected by the rapid rise in population and drastic change in climate. Rice serves as the primary dietary source for half of the world’s population (Hassan et al., 2023). Though rice is grown globally, Asian countries contribute over 50% of the global rice production with 25% of production primarily from rainfed ecosystems (Naresh et al., 2011; Hassan et al., 2023). The rapidly growing population necessitates the need to double the world’s rice production to ensure food security for the anticipated 9.7 billion people by 2050, which is a big challenge under the adverse effects of climate change (Rahman et al., 2022; Chengqi et al., 2023). Under the changing climatic scenario, drought has emerged as the most critical factor limiting crop productivity in rainfed ecosystems resulting in an annual yield loss of about 13 to 35% (Muthu et al., 2020). Global warming and erratic rainfall patterns have intensified the occurrence of dry spells in arid and semi-arid regions, affecting nearly 23 million hectares of rainfed rice cultivation (Pandey and Shukla, 2015; Hussain et al., 2021). In India where 50% of rice is grown under rainfed conditions, climatic variability has increased drought-prone areas by 1.3% (Singh et al., 2017; Krishnan et al., 2021).

Severe drought has shown adverse effects on plant growth, physiology and reproduction (Barnabás et al., 2008; Fahad et al., 2017). Unlike other crops, rice is mostly cultivated under flooded condition, making it more vulnerable to drought stress (Panda et al., 2021). Generally, the response of a rice plant to drought stress varies owing to its varying water requirements at different growth stages (early seedling, vegetative and reproductive) and the severity of stress experienced during each phase. Drought stress during the early seedling stage, leads to poor crop establishment (Vibhuti et al., 2015), while during the vegetative stage, it results in delayed panicle initiation, ultimately declining yield by 21 to 50.6% (Zhang et al., 2018). A short span of drought stress during flowering has adverse effects on pollination, causing poor seed setting, reduced grain size and grain number and in severe cases flower abortion, resulting in a 100% yield decline (Hassan et al., 2023). Even moderate to severe drought stress during the reproductive growth phase can cause substantial yield loss ranging from 51% to 90.6% (Zhang et al., 2018). A meta-analysis by Zhang et al. (2018) has proved that rice is highly sensitive to drought during the reproductive stage (*viz*., blooming, grain filling and maturity), as any intensity of drought stress (mild or severe) can decline rice grain production due to reduced translocation of photo-assimilates from source (leaves) to sink (reproductive organs). Therefore, developing cultivars with reproductive stage drought tolerance is imperative to stabilize grain yield while mitigating the adverse effects of drought stress.

Since the advent of modern rice breeding, achieving high grain yield has remained the primary objective. Over time, efforts led by the International Rice Research Institute (IRRI) have demonstrated the effectiveness of direct selection for grain yield under drought over secondary traits in the Rainfed Rice Breeding program (Venuprasad et al., 2007; Kumar et al., 2008; Khanna et al., 2022). However, enhancing drought tolerance remains a substantial challenge owing to the complex nature of grain yield under drought stress; mainly due to the involvement of small and large effect genes, their epistatic interactions, as well as interactions with the environment and other abiotic stresses.

Genotype (G), Environment (E) and their interaction (G×E) exists widely in nature. G×E interaction (GEI) often leads to differential responses of genotypes across growing environments, resulting in a biased assessment of the genotype effect (Bose et al., 2014). Therefore, assessing the significance of the genetic and environmental factors is crucial in the selection process involving low heritable traits due to more pronounced GEI (Malosetti et al., 2013; Olivoto et al., 2019). The recent trends in plant breeding, amidst a rapidly changing climate, underscore the importance of stability and adaptability of cultivar performance under diverse environmental conditions (Hall and Richards, 2013; Li et al., 2019). Multi-environment trials (METs) are frequently carried out to examine the G×E interaction and to identify the most effective and consistent cultivars across a range of environments. Generally, MET analysis is predominantly utilized to evaluate the impact of drought stress on crop yield (Khanna et al., 2022; Lee et al., 2023). Breeders employ a range of statistical tools to identify stable cultivars for the specific target environments. In recent times, linear mixed-effects models (LMM) have become increasingly popular. Best Linear Unbiased Prediction (BLUP) is widely regarded as a preferred method due to its ability to provide greater precision across diverse experimental conditions. It maximizes the correlation between true and predicted genotypic values, which is crucial for breeders (Furlani et al., 2005; Pathy and Mohanraj, 2021). BLUP estimates are frequently observed to be more precise than BLUE (Best Linear Unbiased Estimates) derived through fixed effect models.

Breeding climate-resilient cultivars underscores the importance of identifying stable potential donors. Plant genetic resources are the key components to meet the future food security needs of the world. Traditional landraces of crops present an excellent source of desirable alleles for the traits of interest, given the advertent and inadvertent selection pressures imposed on them over a long period of time. Generally, traditional landraces are extensively cultivated in rainfed conditions across India. Despite their less productive nature, they possess excellent growth adaptation features to a wide range of stresses. India, being the primary center of origin for rice, possesses rich biodiversity with more than 2,00,000 rice varieties out of which more than 10,000 popular traditional rice accessions have been cultivated throughout India since countless centuries (Ashraf and Lokanadan, 2017). However, only a small fraction of the existing landraces has been used in practical breeding. Therefore, the possibility of identifying potential donors for reproductive stage drought tolerance is relevant by understanding the genetic differences residing among these traditional rice cultivars.

Therefore, the present study focused on evaluating a panel of Indian rice germplasm to understand the yield gaps between non-stress, managed stress and natural stress conditions, to differentiate high-yielding drought-tolerant accessions from drought-sensitive accessions for identifying stable high-yielding drought-tolerant accessions for further use as pre-breeding genetic stocks to enhance yield under drought stress.

## 2 Materials and methods

### 2.1 Plant material

The present study involves a collection of 500 diverse rice germplasm accessions (**Supplementary Table 1**) from 23 states across India covering a wide range of climatic zones. Also, the seven reference checks, consisting of four known drought-tolerant entries (Apo, Norungan, CO 53 and Anna (R) 4) and three drought-susceptible entries (IR 64, Jaya and Pusa 44) were included. The seeds of these accessions were obtained from the Indian National Gene Bank, National Bureau of Plant Genetic Resources (NPBGR), New Delhi.

### 2.2 Multi environment trial

To explore the characteristic differences among the germplasm, these 500 accessions were evaluated in three distinct environments *viz.*, non-stress or irrigated condition (Control), managed stress condition (Rainout shelter, ROS) and natural stress condition (Target Population of Environment, TPE). The experiment was laid out in an Augmented Randomized Complete Block Design (ARCBD) with seven reference checks being replicated in 10 blocks. All the recommended cultural practices were implemented for better crop establishment and crop stand.

#### 2.2.1 Phenotyping under irrigated condition

**Figure 1:**
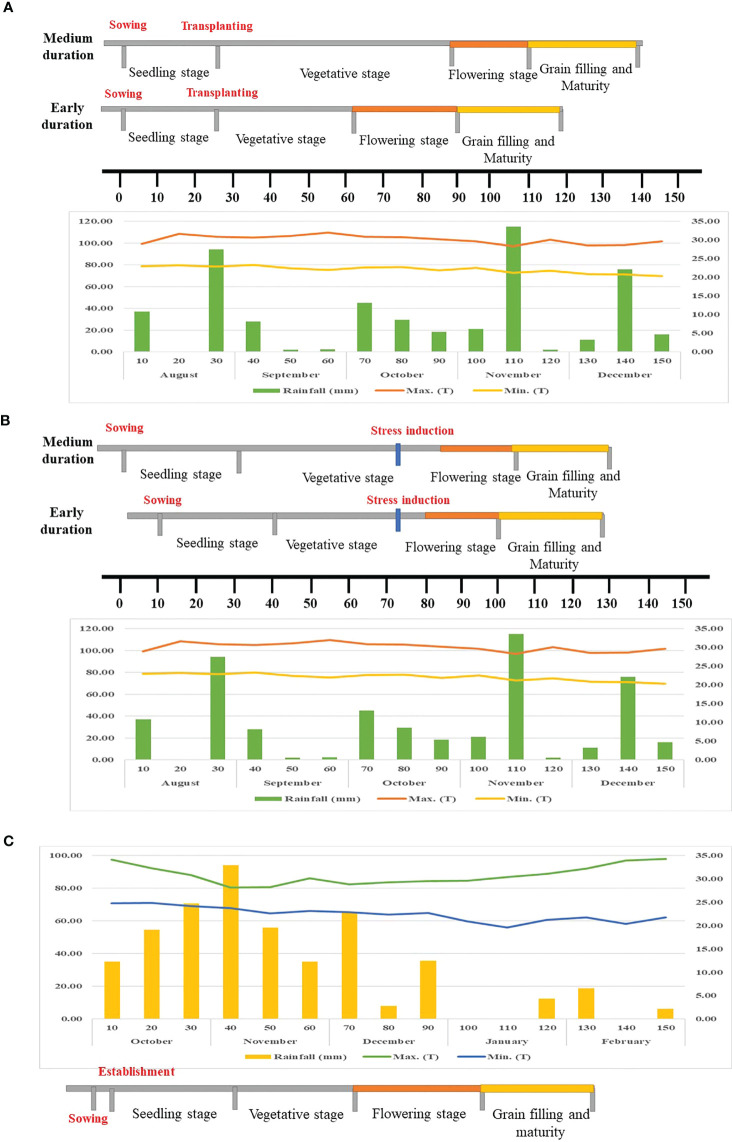
Weather parameters recorded during the growth stage. **(A)** Non-stress condition, **(B)** Managed stress condition **(C)** Natural stress condition.

For evaluation in irrigated or non-stress conditions, the experiment was carried out under flooded, puddled, transplanted conditions in a typical lowland area at Paddy Breeding Station (PBS), TNAU, Coimbatore (latitude 11° N, longitude 77° E) during *Kharif* 2022. The experimental plot consisted of two rows, each measuring 2.6 m in length, with a row-to-row and plant-to-plant spacing of 20 cm. Flooded conditions (maximum 5 cm water depth) were maintained throughout the growth period by adding supplemental irrigation whenever the water depth reduced to 1 cm. The weather data was obtained from the Department of Agrometeorology, Tamil Nadu Agricultural University (TNAU), Coimbatore, India (**Figure 1A**). During the growth period, the amount of rainfall measured under non-stress condition was 497.70 mm, with an average maximum temperature of 30.12°C and minimum temperature of 22.08°C.

#### 2.2.2 Phenotyping under managed stress condition

For evaluation in managed stress conditions, the crop was raised under Rainout shelter (ROS) at Paddy Breeding Station (PBS), TNAU, Coimbatore (latitude 11° N, longitude 77° E) during *Kharif* 2022. The experimental plot consisted of two rows, each measuring 1.2 m in length, with a row-to-row and plant-to-plant spacing of 20 cm. The experiment was carried out under direct-sown puddled conditions in leveled fields representing a lowland environment. The field was flooded until the vegetative stage by maintaining a water level of 5 cm. The drought stress was imposed during the reproductive stage by draining the water at the booting stage and withholding irrigation until harvest (**Figure 1B**). The intensity of drought stress was based on tensiometer readings and observations of wilting and leaf rolling in susceptible checks, which generally occurred about 5–7 days after the tensiometer registered a SWP value of −90 kPa. The weather data was obtained from the Department of Agrometeorology, TNAU, Coimbatore, India (**Figure 1B**). During the growth period, the amount of rainfall measured under managed stress conditions was 497.70 mm, with an average maximum and minimum temperature of 30.12°C and 22.08°C, respectively.

#### 2.2.3 Phenotyping under natural stress condition

For evaluation in natural stress condition, the crop was raised under Target Population of Environment (TPE) at Agricultural Research Station (ARS), Paramakudi (latitude 9° 21’ N, longitude 78° 22’ E) during *Rabi* 2022-2023. The experimental plot consisted of two rows, each measuring 3 m in length, with a row-to-row spacing of 20 cm. The experiment was conducted under direct-sown, non-puddled, non-flooded conditions in leveled fields under rainfed conditions. The entire crop growth period was purely dependent on seasonal rainfall. The weather data was obtained from the Meteorological observatory (TNAU) in Paramakudi, India (**Figure 1C**). The maximum temperature varied from 25.87°C to 36.48°C, while the minimum temperature ranged from 18.67°C to 25.94°C. The amount of rainfall received during the growth period under natural stress condition was 491.62 mm (October 2022 to February 2023). The minimum rainfall of 64.55 mm during the flowering and grain-filling phase (December 2022 to January 2023) indicates the severity of drought and justifies the need for experimentation and development of drought tolerant varieties for these regions.

### 2.3 Traits assessed

Data on yield and yield attributing parameters were recorded in each environment, from five randomly selected plants from the middle of the rows in each of the accessions. Days to fifty per cent flowering (DFF, days) was computed as the duration from sowing to the emergence of the panicle in fifty per cent of the plants within a row. Plant height (PH, cm) was measured from the surface of the soil to the tip of the primary panicle. Tillers per plant (TP) was obtained by counting the total number of tillers per plant on each hill. Number of productive tillers (NPT) was obtained by counting the number of panicles bearing tillers on each hill. Panicle exsertion (PE) was visually scored using the standard evaluation system (SES) for rice (IRRI, 2013). Panicle length (PL, cm) was measured on the primary panicle from its base to the tip. Panicle weight (PW, g) was recorded by measuring the weight of the primary panicle. Hundred seed weight (HSW, g) was obtained by weighing 100 randomly selected filled grains per plant. Spikelet fertility (SF, percentage) was calculated as the ratio of filled grains per panicle to the total number of grains per panicle. The grain yield for each entry (GY, t ha^-1^) was calculated based on the grain weight obtained on a plot basis, standardized to a moisture content of 13% and converted to the hectare scale.

Drought sensitivity in managed stress conditions (ROS) was assessed using leaf rolling and lead tip drying scores based on the standard evaluation system (SES) for rice (IRRI, 2013). Physiological parameters such as relative water content and Chlorophyll Content Index were assessed under control and managed stress conditions (ROS). Relative water content (RWC) was determined on the flag leaf from three different plants, following the method outlined by Turner (1981). Similarly, the Chlorophyll Content Index (CCM) was measured on the same leaves using a Chlorophyll Content Meter (CCM) (CCM-200 *plus*, OPTI-SCIENCE, INC).

### 2.4 Statistical analysis

The overall analysis was carried out as a two-step process. In the first step, referred to as Single trial analysis, the BLUE values were calculated for genotypes for every individual experiment to adjust for the experimental design factors. Whereas in the second step, referred to as Multi Trial Analysis, the genotypes were considered as random effects to compute the BLUP values for all the genotypes correcting for the environment effect.

#### 2.4.1 Single trial analysis

The phenotypic data obtained from three distinct environments *viz*., non-stress condition, managed stress condition and natural stress condition were subjected to single trial analysis (STA) using the package LMMsolver (Boer, 2023) in R software. The best linear unbiased estimator (BLUE) values were computed for all the yield, yield attributing and physiological traits in the single trials where the *genotype* and *rep* were modeled as fixed and random effects, respectively.


$$Y_{raw}=Genotype\;+\;Rep\;\left(checks\right)+\;\epsilon$$

Broad sense heritability (H^2^) was calculated to estimate the quality of the trial in an individual environment, using the formula, H^2^ = vg/(vg + ve), where vg is the genetic variance and ve is the error variance. The Pearson correlations among traits and environments were estimated based on the BLUEs using the “corrplot” package in the R software (Wei et al., 2017).

#### 2.4.2 Multi trial analysis

The BLUE values from the single trial analysis were used as the input for the multi-trial (multi-environment) analysis using the package sommer (Covarrubias-Pazaran, 2016). The best linear unbiased estimator (BLUP) is computed across all single trials with *genotype* and *environment* modeled as random and fixed effects, respectively.


$$Y_{BLUP}=Genotype\;+\;Environmet+\;\epsilon$$

Multi-environment trait correlation was computed based on BLUP of yield and yield-related traits using the “corrplot” package in the R software (Wei et al., 2017).

Stepwise multiple regression analysis was done with the grain yield from the multi-environment analysis as the dependent variable and all phenotypic traits that are moderately or highly correlated with the grain yield as independent variables. The lm function in R software was used to perform the multiple regression analysis (R Core Team, 2021).

#### 2.4.3 Multi-trait selection index

The selection index method was used to select accessions based on more than one trait of interest. The weights for the individual traits were determined using the desired gain approach (Pešek and Baker, 1969) using the formula below,


$$G^{-1}\;d\;=b$$

where a *G* is a n x n covariance matrix; *d* is an n x 1 vector of desired standard deviation and *b* is a n x 1 vector of computed weight for all the traits of interest. In this study, scaled values of the traits were used, aimed to improve one standard deviation and half standard deviation for grain yield and spikelet fertility respectively. The days to fifty per cent flowering was intended to be fixed and therefore it was set to zero standard deviation.

## 3 Result

### 3.1 Single trial analysis

The characteristic differences residing among the 500 Indian rice germplasm for yield and yield attributing traits were assessed in three distinct environments *viz.*, non-stress or irrigated condition (Control), managed stress condition (ROS) and natural stress condition (TPE). Environmental conditions such as maximum temperature (°C), minimum temperature (°C) and rainfall (mm) during each growth phase varied across the environments (**Figure 1**).

#### 3.1.1 Effect of drought on yield and yield-attributing traits

Grain yield is an important agronomic trait of economic importance. Drought stress imposed by natural (TPE) or artificial (ROS) means resulted in soil drying, leading to subsequent yield reductions under these stress conditions compared to the irrigated control. The yield reduction in drought-tolerant checks (Apo, Anna (R) 4, CO 53 and *Norungan*) was 36.46% and 64.54% under ROS and TPE conditions, respectively. However, the reduction was much higher at about 76.56% (ROS) and 82.00% (TPE) in drought-sensitive checks (IR 64, Jaya and Pusa 44). Based on the Best Linear Unbiased Estimator (BLUE) values, the reduction in grain yield among the screened accessions under drought stress was 64.30% in ROS and 68.12% in TPE (**Supplementary Table 2**). Based on the ranking of reference checks, the accessions were classified into drought tolerant and susceptible categories (**Supplementary Table 2**). Among them, 7.40% (37 accessions) in ROS and 20.80% (104 accessions) in TPE were found promising with yield superiority over all the drought-tolerant reference checks. Thus, they are categorized as high-yielding and drought-tolerant in their respective environments. Likewise, 29.00% (145 accessions) in ROS [ranking between Apo and Anna (R) 4] and 33.60% (168 accessions) in TPE [ranking between Norungan and Anna (R) 4] were categorized as moderately resistant. Meanwhile, 30.20% (151 accessions) in ROS and TPE were categorized as moderately susceptible, with rankings below Anna (R) 4 but above IR 64. In contrast, 33.40% (167 accessions) in ROS and 15.40% (77 accessions) in TPE had grain yields lower than those of all susceptible checks (ranking below IR 64), thus categorizing them as highly drought-susceptible.

**Figure 2:**
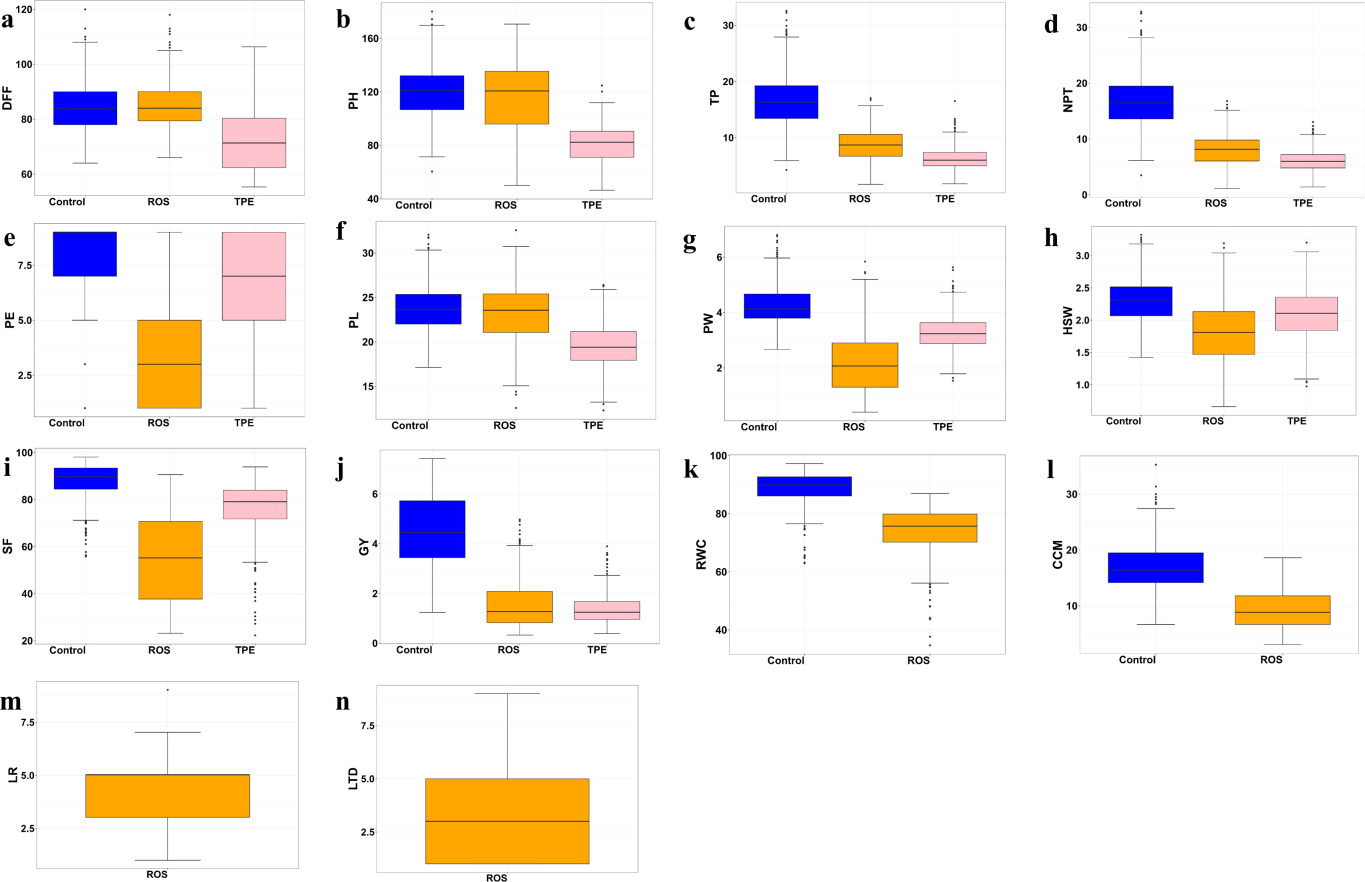
Box plots indicating the distribution pattern of yield and yield-attributing traits across non-stress (Control) and stress environments (ROS, TPE). Shaded regions represent the distribution pattern of the traits. The upper, median and lower quartiles of the boxes represent the 75th, 50th, and 25th percentiles of the accessions, respectively. The horizontal solid bar inside the quartile box represents the mean of the trait and the black dot indicates the outliers. **(a)** DFF - days to fifty per cent flowering, **(b)** PH - plant height (cm), **(c)** TP - tillers per plant, **(d)** NPT - number of productive tillers per plant, **(e)** PE - panicle exsertion, **(f)** PL - panicle length (cm), **(g)** PW - panicle weight (g), **(h)** HSW - hundred seed weight (g), **(i)** SF - spikelet fertility (%), **(j)** GY - grain yield (t ha^-1^), **(k)** RWC – relative water content (%), **(l)** CCM - chlorophyll content index, **(m)** LR - leaf rolling, **(n)** LTD - leaf tip drying.

The yield reduction under drought environments indicates the difference in response of yield-attributing traits across non-stress and stress environments (**Table 1**). The mean values of all the traits under drought stress (ROS and TPE) were lower than those of the control, indicating the impact of drought stress on the performance of the genotype (**Figure 2**). The distribution pattern of the traits, across environments suggests that the majority of the accessions had values clustered around the mean. The extent of reduction of yield-attributing traits was less under ROS while more under TPE, where plant height was reduced by 3.17 cm and 38.00 cm in ROS and TPE, respectively; tillers per plant and number of productive tillers per plant were reduced by about 7.77 and 8.72 in ROS and 10.25 and 10.71 in TPE; panicle length was reduced by 0.60 cm and 4.44 cm in ROS and TPE, respectively. However, the extent of reduction of panicle exsertion, panicle weight and spikelet fertility was higher in ROS and less in TPE compared to the non-stress condition, which indicates the role of fertility breakdown is higher under ROS than TPE. Early flowering was observed under both stress environments (from 85.26 days in control to 84.78 days and 72.89 days in ROS and TPE, respectively).

**Table 1:** Metrics of yield attributing and physiological traits under three different environments.

Traits	Environment	Mean	Range	Heritability	CV (%)
Days to fifty per cent flowering	Control	85.26	64.00 - 120.00	0.98	10.90
	ROS	84.78	66.00 - 118.00	0.96	10.22
	TPE	72.89	55.34 - 106.34	0.99	15.99
Plant height (cm)	Control	118.99	60.38 - 180.05	0.90	16.41
	ROS	115.82	50.00 - 170.67	0.95	22.32
	TPE	80.99	46.40 - 124.80	0.85	17.52
Tillers per plant	Control	16.63	4.25 - 32.58	0.79	29.65
	ROS	8.86	1.70 - 17.03	0.65	32.46
	TPE	6.38	1.80 - 16.50	0.75	30.87
Number of productive tillers per plant	Control	16.84	3.49 - 32.82	0.80	29.18
	ROS	8.12	1.15 - 16.82	0.74	34.98
	TPE	6.13	1.40 - 13.04	0.76	30.03
Panicle exsertion	Control	7.73	1.00 - 9.00	0.78	26.59
	ROS	3.62	1.00 - 9.00	0.92	76.58
	TPE	6.56	1.00 - 9.00	0.90	38.12
Panicle length (cm)	Control	23.92	17.13 - 32.07	0.51	10.52
	ROS	23.30	12.56 - 32.56	0.66	13.85
	TPE	19.48	12.29 - 26.41	0.67	12.52
Panicle weight (g)	Control	4.28	2.67 - 6.80	0.90	17.75
	ROS	2.16	0.40 - 5.84	0.97	52.37
	TPE	3.29	1.54 - 5.63	0.85	17.98
Hundred seed weight (g)	Control	2.32	1.42 - 3.32	0.92	14.67
	ROS	1.82	0.66 - 3.19	0.95	24.88
	TPE	2.09	0.97 - 3.20	0.91	18.56
Spikelet fertility (%)	Control	87.80	55.79 - 98.08	0.60	8.52
	ROS	54.48	23.14 - 90.60	0.89	35.52
	TPE	76.30	22.30 - 93.90	0.76	14.78
Grain yield (t/ha)	Control	4.57	1.24 - 7.42	0.88	31.47
	ROS	1.55	0.33 - 4.97	0.85	61.14
	TPE	1.37	0.39 - 3.90	0.80	43.06
Relative water content (%)	Control	88.97	62.96 - 97.21	0.55	6.07
	ROS	75.56	34.69 - 86.96	0.73	10.47
Chlorophyll content index	Control	16.95	6.68 - 35.27	0.69	24.81
	ROS	9.24	3.05 - 18.62	0.79	37.38
Leaf rolling	ROS	4.67	1.00 - 9.03	0.69	28.81
Leaf tip drying	ROS	3.15	1.00 - 9.00	0.87	55.55

Control, Non stress condition; ROS (Rainout shelter), Managed stress condition; TPE (Target population of Environment), Natural stress condition; CV, Coefficient of variation (%).

Broad sense heritability (H^2^) was estimated for all the studied traits in each environment (**Table 1**). Heritability estimates of yield and yield attributing traits were high under drought-stress environments with a range of 0.65 to 0.97 in ROS and 0.67 to 0.99 in TPE. Similarly, most of the traits under non-stress environment showed high heritability, except panicle length. The heritability of certain traits was high under stress environments and for some traits, it was high under non-stress environments. Knowledge of the association between grain yield and various agronomic and physiological traits is crucial, as it aids in identifying key pre-breeding traits that can serve as the best indicators of grain yield. Pearson correlation analysis between yield and yield-attributing traits under stress and non-stress conditions revealed that grain yield showed significant positive association with plant height, tillers per plant, number of productive tillers per plant and most of the panicle traits *viz*., panicle length, panicle weight, spikelet fertility and hundred seed weight (**Supplementary Figure 1**). However, days to fifty per cent flowering showed a significant negative correlation with grain yield under ROS.

#### 3.1.2 Effect of drought on physiological parameters

Leaf rolling is one of the drought avoidance mechanisms envisaged to maintain yield under drought conditions. Among the accessions screened at managed stress condition (ROS), 29.00% exhibited a leaf rolling score of 1.00 to 3.03, while 30.60% exhibited a leaf tip drying score of 1.00 (**Supplementary Table 2**). Physiological parameters like relative water content and chlorophyll content index were reduced under ROS compared to control (**Table 1**). Relative water content varied from 62.96% (RL 213) to 97.21% (RL 6366) under control and 34.69% (RL 213) to 86.96% (RL 5120) in ROS (**Supplementary Table 2**). Similarly, the chlorophyll content index ranges from 6.68 (RL 2402) to 35.27 (RL 3879) and 3.05 (RL 2422) to 18.62 (RL 1339) in control and ROS, respectively (**Supplementary Table 2**).

Heritability estimates of physiological parameters (RWC and CCM) were high under ROS, while RWC under control exhibited moderate heritability. Similarly, leaf rolling and leaf tip drying exhibited high heritability (**Table 1**). Understanding the association between yield and physiological traits provides a better insight into the mutual relationship that coexist among them. Under ROS, grain yield showed a significant positive association with leaf tip drying (0.18) (**Supplementary Figure 1**). In addition, leaf rolling and leaf tip drying exhibited a significant positive association (0.47).

### 3.2 Multi-environment analysis

Understanding the association between environments is of prime importance as it helps to identify the performance of genotypes across environments. The correlation between the environments varied from 0.00 to 0.89 across all the traits, indicating the existence of low to high G × E interaction (**Table 2**). Almost all the correlations between the environments were positive. However, the magnitude of correlation varied across different traits. In the present study, correlations above 0.60 were considered high, while below 0.30 is considered low. The traits *viz.*, days to fifty per cent flowering, plant height, hundred seed weight and relative water content exhibited high correlations, indicating the occurrence of low G × E, i.e. the performance of genotypes was similar between environments (Control, ROS and TPE). Likewise, the association between environments were moderate for panicle exsertion, panicle length, panicle weight, spikelet fertility and chlorophyll content index. However, the magnitude of correlation was low for tillers per plant, number of productive tillers per plant and grain yield indicating the occurrence of high G × E, i.e. the performance of genotypes varied across environments.

**Table 2:** Trait correlation between the environments.

Traits	Environment	Control	ROS	TPE
Days to fifty per cent flowering	Control	1.00		
	ROS	0.82	1.00	
	TPE	0.68	0.60	1.00
Plant height	Control	1.00		
	ROS	0.68	1.00	
	TPE	0.74	0.67	1.00
Tillers per plant	Control	1.00		
	ROS	0.22	1.00	
	TPE	0.10	0.21	1.00
Number of productive tillers per plant	Control	1.00		
	ROS	0.21	1.00	
	TPE	0.13	0.20	1.00
Panicle exsertion	Control	1.00		
	ROS	0.34	1.00	
	TPE	0.53	0.26	1.00
Panicle length	Control	1.00		
	ROS	0.62	1.00	
	TPE	0.41	0.20	1.00
Panicle weight	Control	1.00		
	ROS	0.42	1.00	
	TPE	0.64	0.20	1.00
Hundred seed weight	Control	1.00		
	ROS	0.65	1.00	
	TPE	0.89	0.57	1.00
Spikelet fertility	Control	1.00		
	ROS	0.33	1.00	
	TPE	0.88	0.29	1.00
Grain yield	Control	1.00		
	ROS	0.26	1.00	
	TPE	0.38	0.00	1.00
Relative water content	Control	1.00		
	ROS	0.76	1.00	
Chlorophyll content index	Control	1.00		
	ROS	0.45	1.00	

Control, Non stress condition; ROS (Rainout shelter), Managed stress condition; TPE (Target population of Environment), Natural stress condition.

**Figure 3:**
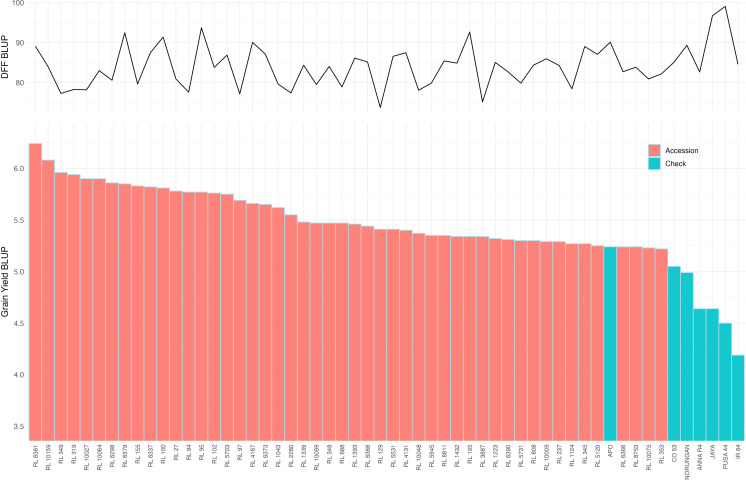
Distribution of best linear unbiased predictor (BLUP) for grain yield from multi-environment trials displayed in the descending. Only the 50 high-yielding accessions are indicated as pink bars and the checks used in the study are represented in Blue bars (Bottom panel). The corresponding days to fifty percent flowering (DFF) for each accession is displayed as a line graph (top panel).

Multi-environment Best linear unbiased prediction (BLUP) values were estimated for all the studied traits (**Supplementary Table 3**). It revealed that 46.35% of the studied accessions had higher grain yield compared with the predicted mean grain yield of 4.58 t/ha (**Supplementary Table 3**). Moreover, 9.40% of accessions (47 accessions) were found promising with yield superiority over all the drought-tolerant checks and hence classified as high-yielding drought-tolerant accessions (**Figure 3**). In contrast, 21.60% of the accessions had predicted grain yields lower than those of all susceptible checks, thus categorizing them as highly drought-susceptible. Among the identified drought-tolerant accessions, 76.60% exhibited a higher spikelet fertility than the predicted mean of 87.82%, with the highest being in RL 4131, RL 1043, RL 6368, RL 4167 and RL 1124 (top 5 accessions). Similarly, 83.00% of the drought-tolerant accessions showed higher panicle weight over the predicted mean (4.26 g) with the highest values in RL 237, RL 1124, RL 4167, RL 155 and RL 6298 (top 5 accessions). Significantly higher panicle length over the predicted mean was noticed in 70.21% of the drought-tolerant accessions. Likewise, 57.45% of the drought-tolerant accessions showed more tillers and productive tillers per plant. Early flowering with less than 85.31 days was observed among 65.96% of the drought-tolerant accessions.

Relative water content varied from 81.64% (RL 213) to 91.47% (Apo), with almost 59.57% of drought-tolerant accessions exhibiting superiority over the predicted mean (89.05%). Similarly, 55.31% of the drought-tolerant accessions exhibited a higher chlorophyll content index than the predicted mean (16.96) with the highest being in RL 1339, RL 808, RL 27, RL 8753 and RL 6361 (top 5 accessions).

#### 3.2.1 Multi-environment trait correlation and regression

**Figure 4:**
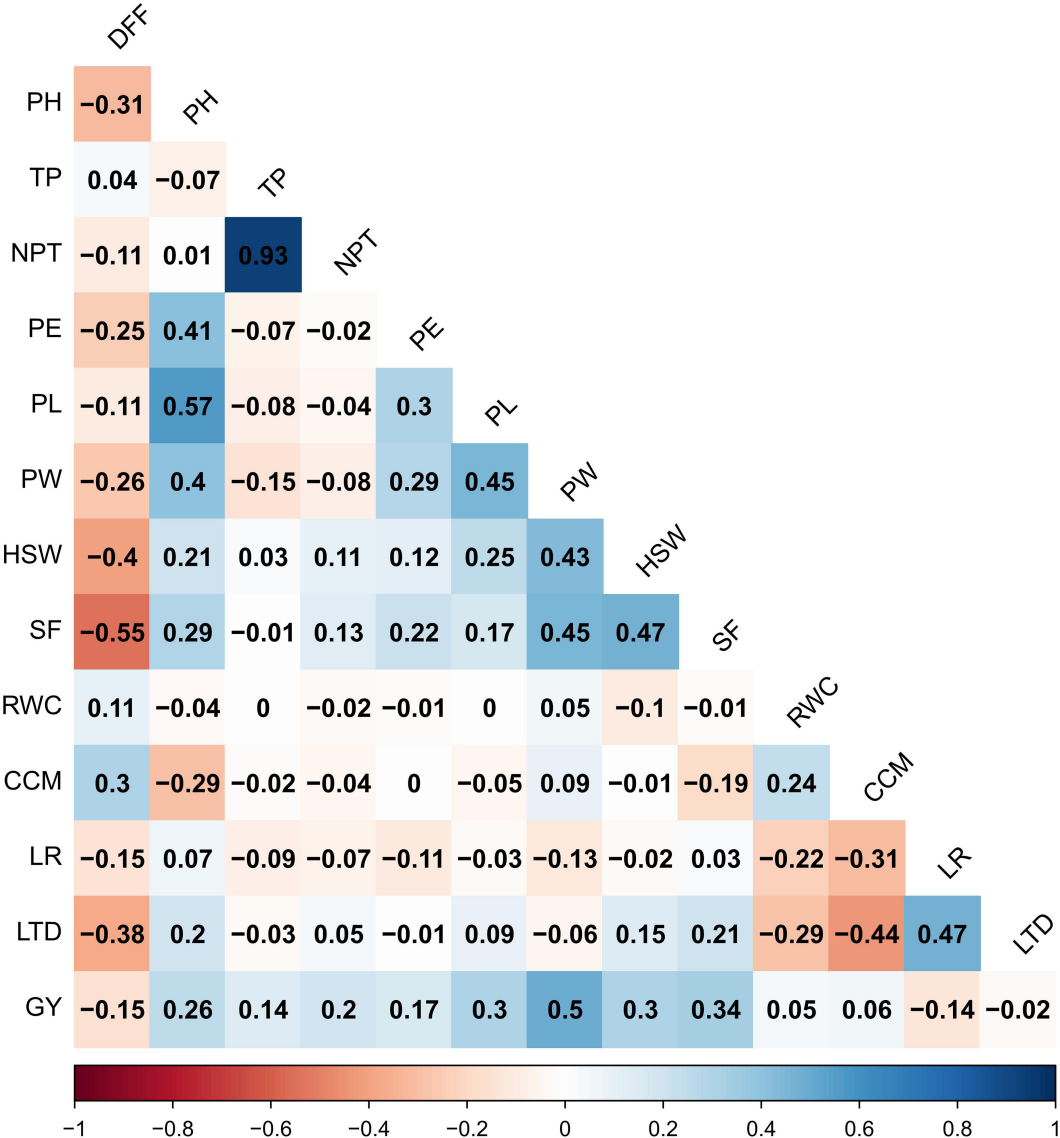
Multi-Environment trait Pearson correlation among the yield and yield-attributing traits. Blue colour indicates a positive correlation and red colour indicates a negative correlation between the traits.

Multi-environment trait correlation using BLUP of yield and yield-related traits revealed that grain yield showed a significant positive association with panicle weight (0.50), spikelet fertility (0.34), panicle length (0.30), hundred seed weight (0.30), plant height (0.26), number of productive tillers per plant (0.20), panicle exsertion (0.17) and tillers per plant (0.14) (**Figure 4**). Similarly, a significant and negative correlation was found between grain yield, days to fifty per cent flowering and leaf rolling.

To identify the most significant traits that influence the yield under both drought and non-stress conditions multiple regression analysis was performed. The grain yield was regressed with multiple traits using stepwise multiple regression, where panicle weight, panicle length, tillers per plant and spikelet fertility were the significant traits that predicted the multi-environment grain yield (r^2^ = 0.325; P< 0.001, **Table 3**).

**Table 3:** Stepwise regression analysis for yield and yield-attributing traits.

Traits	t-value	P-value
Panicle weight	9.537	< 2e-16***
Panicle length	2.217	0.027*
Tillers per plant	6.534	1.59e-10***
Spikelet fertility	2.822	0.005*
Multiple R^2^: 0.325, Adjusted R^2^: 0.320 F-statistic: 59.67 on 4 and 495 DF, p-value:< 2.2e-16		

*** 0.1% level of significance; ** 1% level of significance; *5% level of significance.

#### 3.2.2 Multi-trait selection index

**Figure 5:**
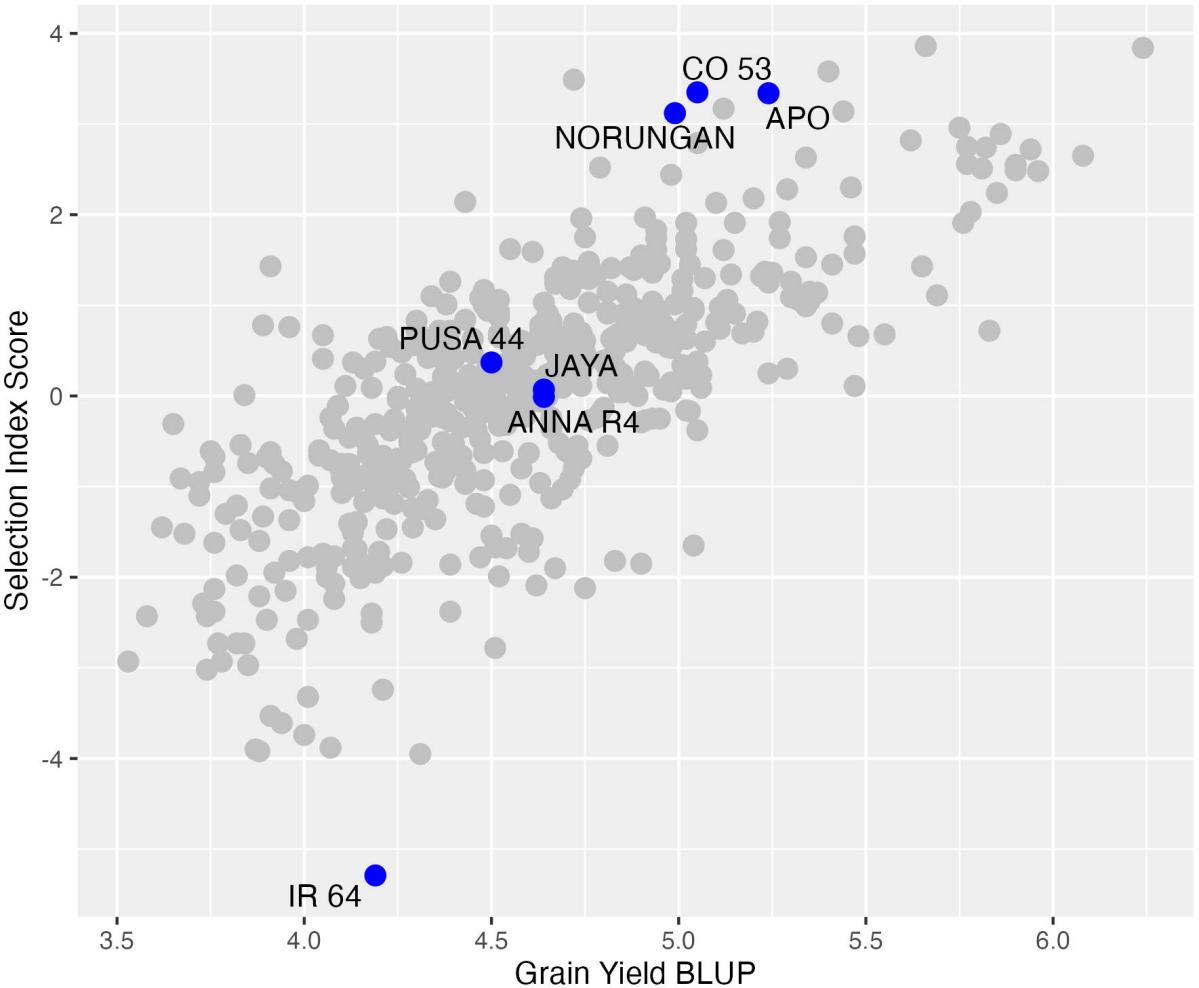
The distribution pattern of the multi-environment BLUP values (x-axis) and the multi-trait selection index scores (y-axis). The blue dot represents the checks and all the best-performing accessions identified using the selection index scores alone are labeled with the lines.

Selection index method was used to select more than one trait of interest to achieve gains in multiple traits. Based on the multi-environment trait correlations and multiple regression analysis a selection index score was computed using grain yield, spikelet fertility and days to fifty per cent flowering. Grain yield and spikelet fertility were subjected to one standard deviation increase while days to fifty per cent flowering was subjected to no increase or decrease. Although days to fifty per cent flowering was not a significant trait influencing the grain yield from the multiple regression analysis, it was still used in the selection index. The selection index scores indicated four accessions (RL 4167, RL 6361, RL 4131 and RL 1650) better than any drought-tolerant checks used in this study (**Figure 5**; **Supplementary Table 4**).

## 4 Discussion

Erratic rainfall distribution in rainfed areas and declining groundwater levels in irrigated areas pose a significant threat to global rice production (Venkateshwarlu et al., 2022). This emphasizes the need for the development of drought-tolerant rice cultivars that could yield more with less water as it could reduce production risks in rainfed regions, alleviate poverty and boost productivity in rice-growing areas amid changing climate conditions. For decades, traditional farmers have cultivated numerous landraces, despite their low-yielding capacity. Modern breeding strategies focus on elite x elite crosses for developing high-yielding cultivars and often ignore the need for landraces in breeding programs. However, the underutilized landraces are the hidden source of valuable tolerant genes/QTLs, which could enhance drought stress tolerance in elite modern breeding cultivars. Since, the crop germplasm serves as a valuable source of genetic diversity (Ahmad et al., 2022b), this study focused on evaluating a panel of Indian rice germplasm to differentiate high-yielding drought-tolerant cultivars from drought-sensitive cultivars and to understand the yield gaps under non-stress, managed stress and natural stress conditions for selecting high yielding stable drought-tolerant accessions.

Yield reduction is a common phenomenon under reproductive stage drought stress as indicated by many earlier studies (Zhang et al., 2018; Bhandari et al., 2020; Ahmad et al., 2022b; Venkateshwarlu et al., 2022). Understanding the mechanisms and responses of genotypes under drought stress is crucial for identifying drought tolerance accessions with high yield potential or those capable of maintaining stable yields under drought conditions. In this study, under both stress environments, namely managed stress condition (ROS) and natural stress condition (TPE), the grain yield of the accessions was reduced compared to the non-stress condition, indicating the impact of drought stress on the performance of the genotype. The reduction in grain yield of about 64.30% under ROS and 68.12% under TPE underscores the severity of drought in stress environments. The yield reduction might be due to the severe reduction in the production and translocation of photo-assimilates from leaves (source) to reproductive organ sink) (Rehman et al., 2002; Hassan et al., 2023). Despite the reduction in grain yield, 7.40% of accessions under managed stress conditions and 20.80% of accessions under natural stress condition were found promising with yield superiority over all the studied drought-tolerant reference checks. Therefore, these accessions are categorized as high-yielding and drought-tolerant in the respective environments. Notably, RL 27, RL 94, RL 97, RL 155, RL 319, RL 349 and RL 6298 were identified as high-yielding and drought-tolerant under both the stress environments (ROS and TPE), indicating their consistency across environments. The promising high-yielding accessions identified in the respective environments could further be evaluated across diverse environments (rainfed, irrigated, and extreme drought scenarios) and varying conditions, such as different soil types and seasons, to identify stable, high-yielding drought-tolerant donors for developing climate-resilient cultivars.

Besides grain yield, major yield-attributing panicle traits like spikelet fertility and panicle weight exhibited substantial reduction under drought stress compared to the non-stress condition. However, the extent of reduction was higher in ROS than TPE, indicating the fertility breakdown is higher under ROS, which might be attributed mainly due to incomplete panicle exsertion. Thus, the reduction in spikelet fertility can be considered a major indicator of yield reduction under drought stress in rice (Swamy et al., 2017; Bhandari et al., 2020; Venkateshwarlu et al., 2022). In this study, a reduction in spikelet fertility of 33.32% and 11.50% in ROS and TPE might have resulted in a substantial yield reduction. This underscores the critical role of water availability during the flowering stage, as inadequate water can impair pollen viability, subsequently reducing the number of filled grains per panicle (Ahmad et al., 2022a, b).

Drought stress affects various physiological processes in plants, triggering several responses that help them to adapt to challenging environmental conditions (Serraj et al., 2009). Leaf tip drying is an early indicator of dehydration and oxidative stress in plants. Among the accessions screened at managed stress conditions (ROS), 29.00% exhibited a leaf rolling score of 1.00 to 3.00, while 30.60% exhibited a leaf tip drying score of 1.00. Among the high-yielding drought-tolerant accessions, RL 27, RL 155, RL 614, RL 6298, and RL 8601 exhibited minimal leaf rolling and leaf tip drying, suggesting their ability to maintain better water status and withstand drought stress with reduced physiological damage. Relative water content (RWC) plays a crucial role in assessing plants dehydration tolerance as it reflects metabolic activity in the tissues. The percentage reduction in RWC content under managed stress condition ranged from 4.19% to 51.27%, with an average reduction of 16.31% compared to the control. Among the high-yielding drought-tolerant accessions identified, RL 614, RL 681, RL 688, RL 808, RL 1230, RL 1393, RL 2294, RL 2342, RL 4167 and RL 8601 exhibited a lower reduction percentage compared to the control, indicating their inherent drought tolerant ability is mainly due to the maintenance of internal water balance under drought-stress situations. Similarly, chlorophyll is a key component of photosynthesis in green plants and is positively associated with the photosynthetic rate. Chlorophyll content and plant responses to tissue water potential determine their level of drought tolerance (Kumar et al., 2016). Under managed drought stress condition, the average reduction in the chlorophyll content index was 44.60%, with a more pronounced reduction observed in drought-sensitive cultivars. This decrease might be attributed to oxidative stress damage caused by chlorophyllase enzymes (Herzog, 1986). Notably among the high-yielding drought-tolerant accessions identified, RL 1393 and RL 27 exhibited a lower reduction in chlorophyll content, suggesting their drought tolerance ability could be due to their ability to maintain higher chlorophyll levels even under water-deficient conditions. Nithya et al. (2020) reported that accessions with high relative water content and chlorophyll stability index could yield high under drought stress. Therefore, these drought-tolerant accessions could serve as valuable genetic resources for pre-breeding programs aimed at developing climate-resilient rice varieties.

Multi-environment trials are often preferred in plant breeding programs as they enhance the accuracy of selecting genotypes by assessing the presence of genotype-by-environment interactions (GEI) (Malosetti et al., 2013). In recent decades, among the statistical methods employed to model GEI, Best linear unbiased prediction (BLUP) has been recognized as one of the most accurate methods for estimating random genetic effects in linear mixed models (Piepho, 1994; Postma, 2006). BLUP helps to compare the performance of genotypes across environments while simultaneously correcting for environmental effects and allows the prediction of real genotypic effects (Bernardo, 2020). Therefore, multi-environment BLUP combining control, ROS and TPE was performed for all the studied traits to identify stable high-yielding drought-tolerant accessions. Similarly, Mahadevaiah et al. (2021) utilized multi-environment BLUP to identify stable, high-yielding, drought-tolerant sugarcane clones.

Multi-environment analysis revealed that 9.40% of the studied accessions (47 accessions) were found promising with yield superiority over the drought-tolerant checks and therefore identified as stable high-yielding drought-tolerant accessions. However, the majority of the high-yielding drought-tolerant accessions identified under multi-environment analysis were the germplasm collections from the Eastern (25% of accessions) and North Eastern regions (30% of accessions) of India. Since the Eastern region of India constitutes about 70% of the rainfed rice-growing areas (Pathak et al., 2020), the evolution of rice cultivars in these drought-prone environments has likely led to the development of numerous drought-tolerant land races that could withstand varying degrees of drought stress. Therefore, the promising high-yielding accessions (47 accessions) identified under multi-environment analysis could be constituted as a working core for drought tolerance and can be evaluated further under various agro-climatic conditions to understand the underlying mechanisms of drought tolerance. This could facilitate targeted breeding, accelerating the development of high-yielding, drought-resilient rice varieties for sustainable agriculture. Notably, the accessions RL 6361, RL 10159, RL 349 and RL 319 were found promising with 30% yield superiority over the predicted mean grain yield. Among the high-yielding drought-tolerant accessions identified, RL 6361, RL 27, RL 6298, RL 6337, RL 349, RL 4131 and RL 1339 exhibited superiority over the mean predicted value for most of the traits (Top 7 accessions). Therefore, these drought-tolerant accessions could be employed as potential donors for developing pre-breeding genetic stocks that could mitigate the adverse effects of climate change. The high-yielding ability in RL 6361, RL 27, RL 6298 and RL 4167 is mainly attributed to better grain-filling as indicated by better panicle exsertion, higher panicle weight and spikelet fertility percentage. In addition, the ability of RL 1393 to maintain relative water content under drought stress indicates its ability to prevent membrane damage. Thus, the high-yielding ability of these accessions could be attributed to their ability to maintain their internal water balance under drought stress conditions. Larbi and Mekliche (2004) reported that accessions capable of maintaining high RWC during the grain-filling phase are potential candidates for ensuring yield stability in semi-arid regions.

Among the high-yielding, stable drought-tolerant accessions, 14 were identified as drought-tolerant under managed stress conditions, while 33 were recognized as drought-tolerant under natural stress condition. Meanwhile, four accessions, *viz*., RL 27, RL 97, RL 155 and RL 6298 were identified as high-yielding and drought-tolerant under both stress environments (ROS and TPE), indicating their consistency across environments. Thus, these high-yielding drought-tolerant accessions can be targeted for regions prone to erratic rainfall or semi-arid rice-growing areas to ensure food security in climate-vulnerable zones. In addition, a strong positive and highly significant correlation between the predicted grain yield and yield under managed stress conditions (0.59, data not shown) and natural stress condition (0.73, data not shown) confirms the reliability of selected high-yielding, stable drought-tolerant accessions. Similarly, Ndikuryayo et al. (2023) examined the relationship between yield under drought stress, yield BLUP and Stress Tolerance Index (STI) for yield and concluded that BLUP can be employed in identifying superior drought-tolerant accessions as yield under drought stress and yield BLUP are highly correlated.

In order to select multiple traits breeders use independent culling, tandem selection and some sort of selection index. Among all, selection index method is advantageous when a breeder needs to improve more than one trait simultaneously without losing the gain for any of the individual traits. In this study, the selection index was computed using grain yield, days to fifty per cent flowering and spikelet fertility with an aim to identify accessions that are high yielding and have higher spikelet fertility but do not increase or decrease in flowering time. Although flowering time is not significant, including flowering time in the index is critical to avoid unconscious selection of accessions that are either late flowering or very early flowering. Multi-trait selection index identified four accessions, namely RL 4167, RL 6361, RL 4131 and RL 1650 with three (RL 4167, RL 6361, RL 4131) of them occurring at the top in both analyses. Identifying stable drought-tolerant donors is imperative to stabilize the grain yield while mitigating the adverse effects of drought stress. Thus, RL 4167, RL 6361, RL 4131 could be employed as potential donors for developing climate resilient cultivars. Furthermore, molecular characterization of the identified stable, high-yielding drought-tolerant accessions based on multi-environment analysis and multi-trait selection index can help identify specific drought-responsive genes, which can be utilized in developing climate-resilient varieties through marker-assisted breeding.

The success of any crop breeding program depends on the knowledge of existing genetic variability, to make the selection more effective. The efficiency of exploiting the genetic variability by selection depends upon the heritability of individual traits. Heritability estimates of yield, yield attributing and physiological traits were moderate to high heritability for all the traits studied. The heritability of certain traits was high under stress conditions and vice-versa. Earlier studies by Swamy et al. (2017); Solis et al. (2018) and Venkateshwarlu et al. (2022) have also reported high heritability estimates for grain yield and yield-attributing traits. High heritability estimate for grain yield (>80%) under both stress and non-stress conditions suggests that selection for grain yield under drought stress in rice can be done with the same precision as in non-stress condition (Kumar et al., 2008; Venkateshwarlu et al., 2022).

Knowledge of the association between grain yield and various agronomic and physiological traits is crucial, as it aids in identifying key pre-breeding traits that can serve as the best indicators of grain yield. Under non-stress condition, the highest correlation was found between grain yield, tillers per plant and number of productive tillers per plant. In contrast, under both stress conditions, the highest correlation was between grain yield, panicle weight and spikelet fertility. This is in accordance with the earlier studies, wherein Liu et al. (2006); Hussain et al. (2021) reported that yield reduction under reproductive stage drought stress is mainly due to increased spikelet sterility and reduced grain weight. Similarly, the multi-environment BLUP correlation revealed that grain yield showed a significant positive association with panicle weight, spikelet fertility, panicle length, hundred seed weight, plant height, number of productive tillers per plant, tillers per plant and panicle exsertion. These results herein show that more tillers might produce more panicles and increased synthesis and translocation of photo-assimilates from the source to sink could significantly improve yield through enhanced grain filling and seed set. The results are in concordance with earlier reports by Hussain et al. (2021); Lee et al. (2023); Ndikuryayo et al. (2023). Similarly, a significant positive association observed between most of the panicle traits like panicle length, panicle weight, hundred seed weight and spikelet fertility emphasizes that an increase in the grain filling rate directly contributes to higher grain yield. Therefore, panicle weight, spikelet fertility and number of productive tillers per plant can be considered as key pre-breeding traits in identifying potential drought-tolerant donors for grain yield improvement under changing climatic scenarios.

## 5 Conclusion

The present study revealed the availability of large genetic variation with high heritability in both drought-stress and non-stress conditions. Our study revealed that a higher drought tolerance potential is mainly attributed to increased panicle weight, spikelet fertility and number of productive tillers per plant. The promising high-yielding accessions (47 accessions) identified under multi-environment analysis could be utilized as a working core for drought tolerance and can be evaluated further under various agro-climatic conditions to understand the underlying mechanisms of drought tolerance. Multi-environment analyses and the multi-trait selection index identified RL 4167, RL 6361 and RL 4131 could serve as potential donors for developing pre-breeding genetic stocks that could mitigate the adverse effects of climate change and enhance productivity in water-limited environments. Further, molecular characterization of the identified stable, high-yielding drought-tolerant donors could identify specific drought-responsive genes, which can be utilized in developing climate-resilient varieties through marker-assisted breeding.

## Data Availability

The original contributions presented in the study are included in the article/**Supplementary Material**. Further inquiries can be directed to the corresponding author.

## Supplementary material

Supplementary Table 1 The list of the rice germplasm accessions used in the study.

Supplementary Table 2 BLUE values of the studied germplasm accession across different environments.

Supplementary Table 3 Multi-Environment BLUP values of the studied germplasm.

Supplementary Table 4 Multi-trait Selection index scores of the studied germplasm.

Supplementary Figure 1 Pearson’s correlation coefficient across environments a) Control b) ROS c) TPE.
