# Supplementary material for: Unraveling the genetic potential of Indian rice germplasm for reproductive stage drought tolerance
Source: Front Plant Sci. 2025 Jul 18;16:1454299. doi: 10.3389/fpls.2025.1454299 (PMC12313633; doi:10.3389/fpls.2025.1454299)

**Supplementary Figure 1. Pearson’s correlation coefficient across environments a) Control b) ROS c) TPE**

**c)**

**a)**

**b)**


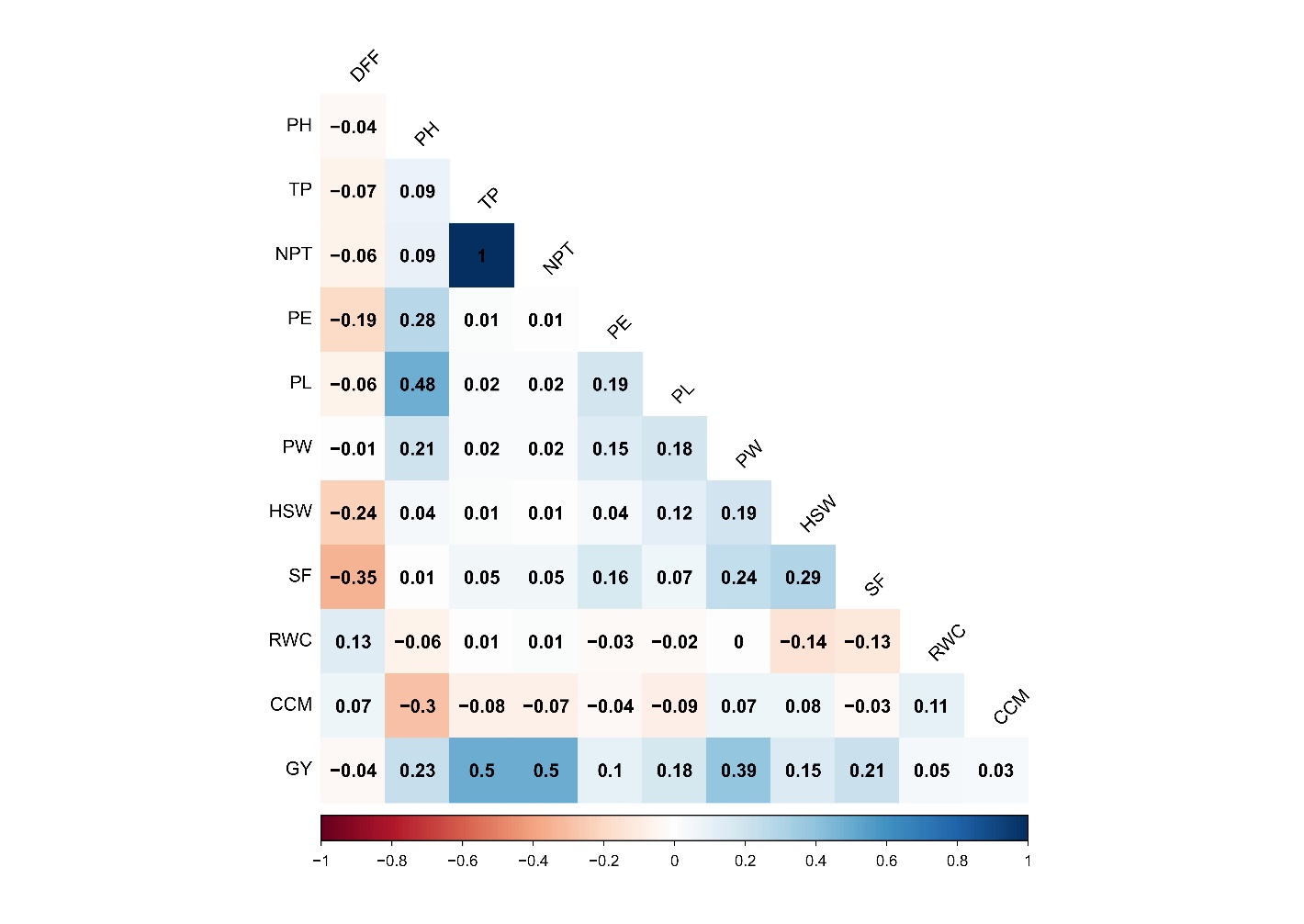


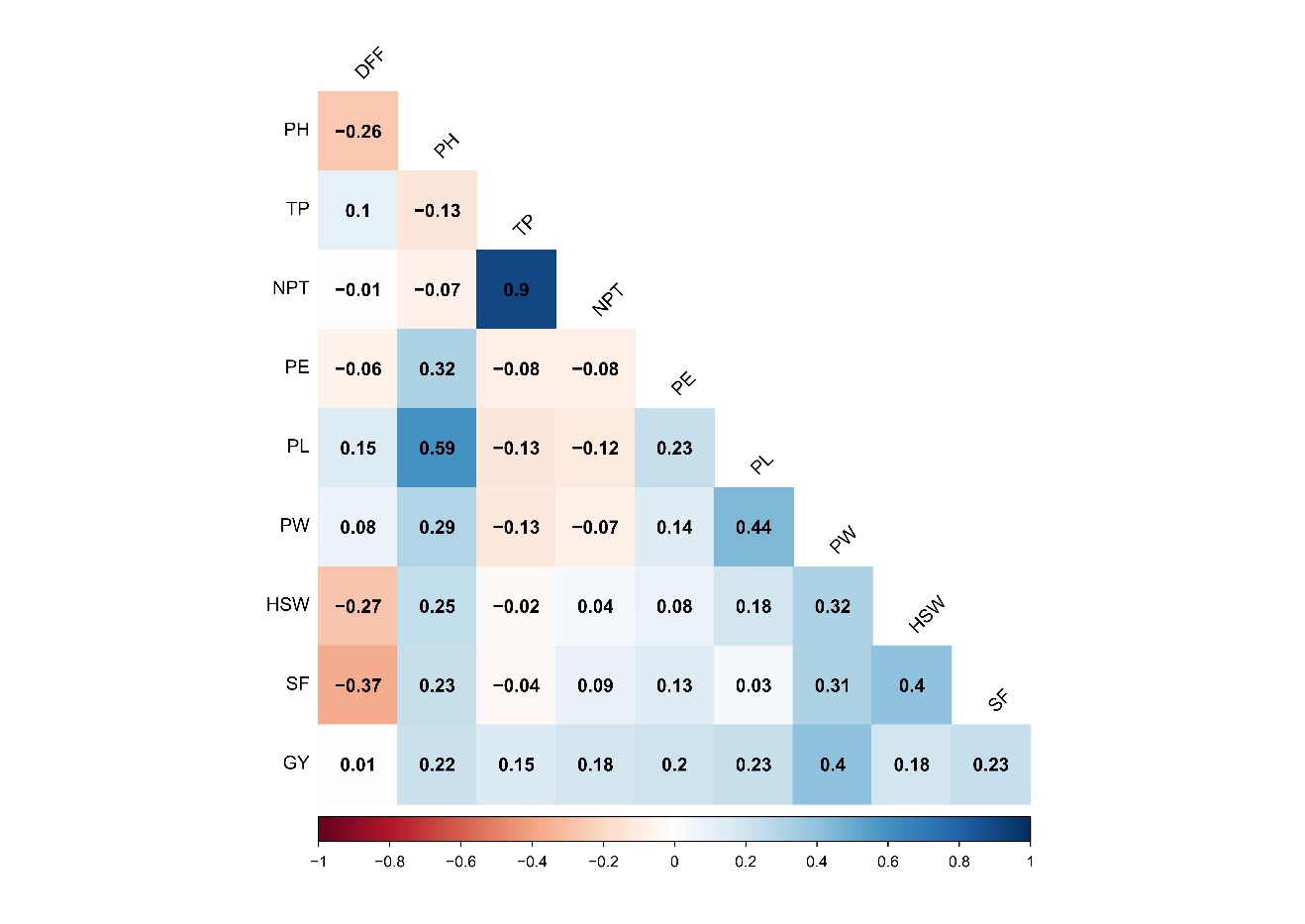

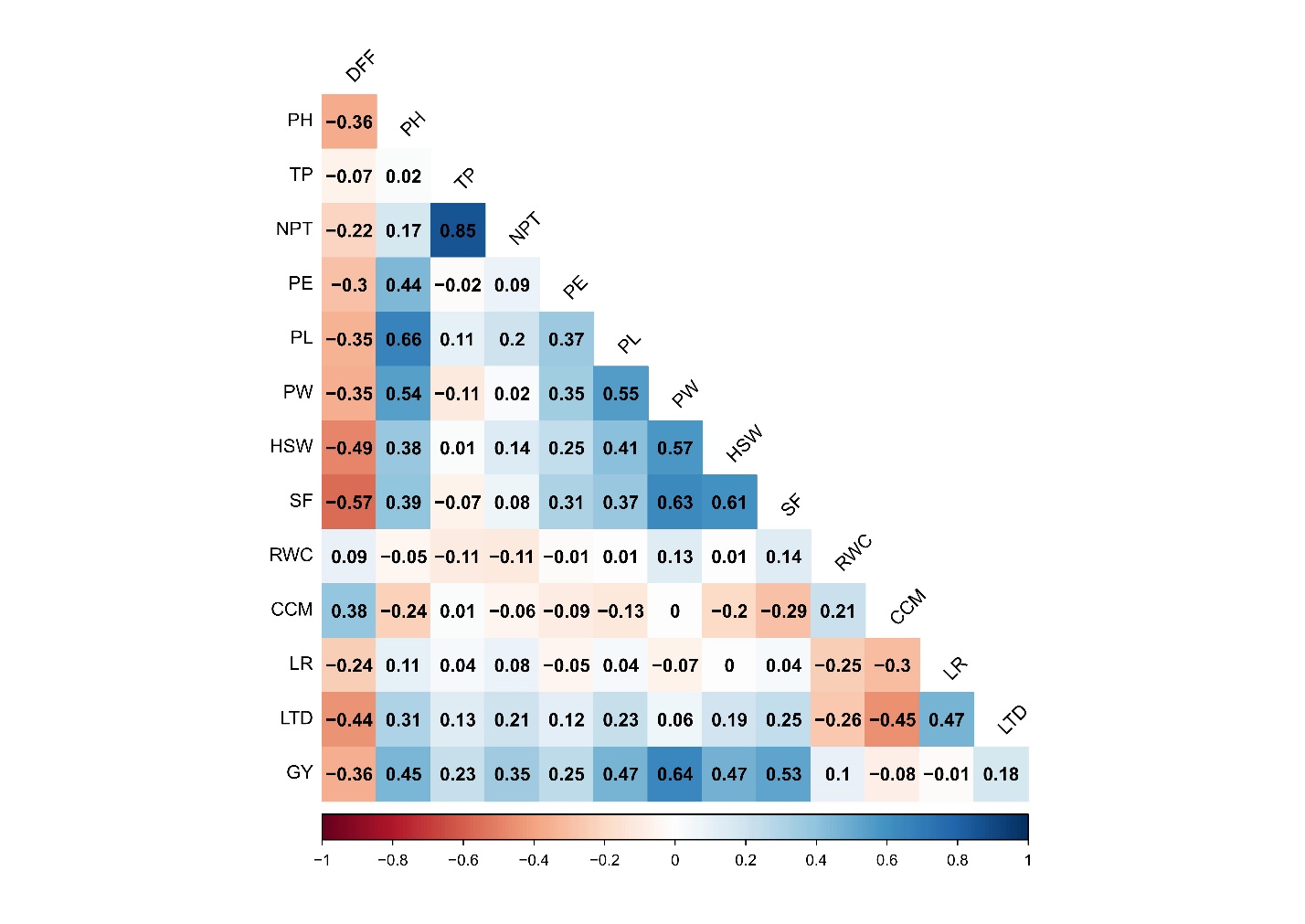

Supplement: Supplementary Table 1 — The list of the rice germplasm accessions used in the study. [file SupplementaryFile1.zip › Supplementary Figure 1.docx]
